# A systematic review and taxonomy of tools for evaluating evidence-based medicine teaching in medical education

**DOI:** 10.1186/s13643-020-01311-y

**Published:** 2020-04-24

**Authors:** Bharathy Kumaravel, Jasmine Heath Hearn, Leila Jahangiri, Rachel Pollard, Claire J. Stocker, David Nunan

**Affiliations:** 1 University of Buckingham Medical School, Hunter Street, Buckingham, MK18 1EG UK; 2 Department of Psychology, Manchester Metropolitan University, Brooks Building, 53 Bonsall Street, Manchester, M15 6GX UK; 3 Department of Life Sciences, Birmingham City University, Birmingham, B15 3TN UK; 4 Franciscan Library, University of Buckingham, Buckingham, MK18 1EG UK; 5 University of Buckingham, Buckingham, MK18 1EG UK; 6 Centre for Evidence Based Medicine, Nuffield Department of Primary Care Health Sciences, Oxford, OX2 6GG UK

**Keywords:** Evidence-based medicine, Competency, Medical education, Assessment

## Abstract

**Background:**

The importance of teaching the skills and practice of evidence-based medicine (EBM) for medical professionals has steadily grown in recent years. Alongside this growth is a need to evaluate the effectiveness of EBM curriculum as assessed by competency in the five ‘A’s’: asking, acquiring, appraising, applying and assessing (impact and performance). EBM educators in medical education will benefit from a compendium of existing assessment tools for assessing EBM competencies in their settings. The purpose of this review is to provide a systematic review and taxonomy of validated tools that evaluate EBM teaching in medical education.

**Methods:**

We searched MEDLINE, EMBASE, Cochrane library, Educational Resources Information Centre (ERIC), Best Evidence Medical Education (BEME) databases and references of retrieved articles published between January 2005 and March 2019. We have presented the identified tools along with their psychometric properties including validity, reliability and relevance to the five domains of EBM practice and dimensions of EBM learning. We also assessed the quality of the tools to identify high quality tools as those supported by established interrater reliability (if applicable), objective (non-self-reported) outcome measures and achieved ≥ 3 types of established validity evidence. We have reported our study in accordance with the PRISMA guidelines.

**Results:**

We identified 1719 potentially relevant articles of which 63 full text articles were assessed for eligibility against inclusion and exclusion criteria. Twelve articles each with a unique and newly identified tool were included in the final analysis. Of the twelve tools, all of them assessed the third step of EBM practice (appraise) and four assessed just that one step. None of the twelve tools assessed the last step of EBM practice (assess). Of the seven domains of EBM learning, ten tools assessed knowledge gain, nine assessed skills and-one assessed attitude. None addressed reaction to EBM teaching, self-efficacy, behaviours or patient benefit. Of the twelve tools identified, six were high quality. We have also provided a taxonomy of tools using the CREATE framework, for EBM teachers in medical education.

**Conclusions:**

Six tools of reasonable validity are available for evaluating most steps of EBM and some domains of EBM learning. Further development and validation of tools that evaluate all the steps in EBM and all educational outcome domains are needed.

**Systematic review registration:**

PROSPERO CRD42018116203.

## Background

Evidence-based medicine (EBM) is the skill of bringing together clinical judgement, the best available evidence from health research along with patient preferences and values in making clinical decisions [1]. EBM involves five steps—asking, acquiring, appraising, applying evidence in clinical decisions and assessing impact and performance [2]. To ensure future medical professionals are better equipped with lifelong skills for evidence-based medicine, we need to ensure that EBM teaching is integrated into undergraduate and postgraduate medical curriculum. In the UK, the General Medical Council recommends that ‘Newly qualified doctors must be able to apply scientific method and approaches to medical research and integrate these with a range of sources of information used to make decisions for care’ (https://www.gmc-uk.org/-/media/documents/dc11326-outcomes-for-graduates-2018_pdf-75040796.pdf).

Researchers have emphasised on the need to shift EBM teaching from the classroom to application of skills in clinical practice to achieve improvement in outcomes [3]. EBM teaching should focus on implementing multifaceted, clinically integrated approaches with assessments of knowledge, skills and behaviour in the medium to long term using validated assessment tools [4]. This highlights the need for validated tools to evaluate the impact of EBM teaching and assessment of medical trainees’ competency.

A systematic review of EBP education evaluation tools in 2006 [5] identified 104 unique instruments for evaluating evidence-based practice (EBP) teaching, though the authors identified only two of them—Fresno [6] and Berlin [7] as high-quality instruments which evaluate knowledge and skills across the EBP steps. The authors defined high-quality instruments as those with established interrater reliability (if applicable), objective outcome measures (non-self-reported) and multiple (≥ 3) types of established validity evidence. They found that among EBP skills, instruments acquiring evidence and appraising evidence were most commonly evaluated, with some newer instruments measuring asking and applying skills. Since the 2006 review, new assessment tools have been developed which assess EBM attitudes and behaviours [8–10].

Despite the availability of tools to evaluate EBM teaching, most evidence-based practice educational interventions still do not use high quality tools to measure outcomes [8]. EBM educators in medical education will benefit by the availability of a compendium of such tools which are classified by their suitability of assessing the five steps of EBM and the various educational outcome domains. Ensuring longitudinal evaluation of EBM teaching using validated assessment tools will provide educators information on the medium to long-term impact of their teaching.

In 2011, a guidance was developed for classification of tools to assess EBP learning, which also recommended a common taxonomy and proposed a framework—CREATE (Classification Rubric for Evidence Based Practice Assessment Tools in Education) for classifying such tools [11]. The purpose of the framework was to help EBP educators identify the best available assessment tool, provide direction for developers of new EBP learning assessment tools and a framework for classifying the tools. To that end, we designed this systematic review to incorporate these updates since the 2006 systematic review to assess and summarise published assessment tools for the evaluation of EBM teaching and learning in medical education.

The primary objective of this review was to summarise and describe currently available tools to evaluate EBM teaching in medical education. We compare, contrast and discuss the tools with consideration given to their psychometric properties and relevance to EBM domains and dimensions of EBM learning. The review aimed to differentiate tools into different subcategories according to type, extent, methods and results of psychometric testing and suitability for different evaluation purposes. The second objective of this review is to produce a taxonomy of tools based on the CREATE framework for medical educators to aid in the evaluation of EBM teaching.

## Methods

### Identification of studies

A scoping search was performed to validate the developed search strategy and justify the importance of conducting a review on the topic as defined by our research question and objectives. This search identified the most recent systematic review on this topic with a search end date of April 2006 [5]. We carried out an initial database search for relevant studies published between Jan 2005 and December 2018 with an update in March 2019.

### Eligibility criteria

We included studies that reported a quantitative and/or qualitative description of at least one tool used to evaluate EBM in medical education which (a) assessed the dimension(s) of EBM learning, namely reaction to educational experience, attitudes, self-efficacy, knowledge, skills, behaviours and benefits to patients and (b) assessed different step(s) of EBM and (c) presented results of the psychometric performance of the tool. In addition to the above criteria, only tools which used objective outcome measures (non-self-reported) were included. We excluded tools which were explicitly designed for use in evaluating EBM teaching for other healthcare professionals (e.g. nurses or dentists). However, if such a tool was later validated for use in medical education, they were included in this review. We only included English language studies. Qualitative studies discussing perceptions of EBM curriculum and did not satisfy the inclusion criteria, conference abstracts, short notes, comments, editorials and study protocols were excluded.

### Search strategy

The following electronic bibliographic databases of published studies were searched: MEDLINE, EMBASE, ERIC, BEME guidelines, Allied and complementary medicine, Cochrane Database of Systematic Reviews (CDSR) and Centre for Reviews and Dissemination (CRD) Databases (Database of Abstracts of Reviews of Effects (DARE). We also searched reference lists of retrieved articles.

### Search terms

Search terms included: ‘Evidence Based Medicine’ or ‘EBM’ or ‘Evidence Based Practice’ or ‘Evidence Based Healthcare’ or ‘Evidence based Health Care’; ‘Educational Measurement’ or ‘assessment tool’; ‘Medical students’; ‘Medical education’; Clinical competence. MeSH terms were supplemented with keywords. Terms were then compared with the indexing terms applied to key journal articles which had previously been identified. An information specialist applied a preliminary search strategy, which was based on medical subject headings (MeSH) terms and text words of key papers that were identified beforehand (see Additional file 1).

### Study selection

The first investigator (BK) carried out initial screening and excluded studies which did not meet the inclusion criteria. This included screening of titles and abstracts to assess their eligibility based on participant characteristics, descriptions of tools, assessment against the five EBM steps and seven educational domains and reporting of psychometric properties of the tools. BK and JHH subsequently screened full text articles against the inclusion and exclusion criteria and any discrepancies were resolved by consensus. When multiple studies presented the evaluation of the same tool, only the first study which evaluated the psychometric properties of the tool in medical education was included in this review, subsequent studies were considered as duplicates.

### Data extraction and analysis

Data extraction was conducted using a standardised data extraction form. Information extracted included type of evaluation tool—description and development of the tool; number, level of expertise in EBM, training level of participants; the EBM steps evaluated; relevance of the tool to the dimensions of EBM learning, namely reaction to educational experience, attitudes, self-efficacy, knowledge, skills, behaviours and benefits to patients and psychometric properties of the tool.

BK and JHH independently reviewed and extracted data, and a third reviewer (LJ) also independently verified the findings of BK and JHH. Results were compared to achieve consensus. Disagreements during data extraction were resolved by consensus. Reviewers were not blinded to any portion of the articles.

BK, JHH and LJ evaluated the quality of each tool using the method from a previous systematic review [5]. Quality was assessed using guidance published by Shaneyfelt et al: (i) established interrater reliability (if applicable), (ii) type of outcome measure and (iii) validity [5]. A tool was rated high quality when supported by established (interrater reliability (if applicable), use of objective (non-self-reported) outcome measure(s) and when it also demonstrated multiple (≥ 3) types of established validity evidence (including evidence of discriminative validity)). Results of quality assessments were compared, and any discrepancies were resolved by consensus.

We first classified included tools and instruments according to the steps of EBM practice and educational outcome domains evaluated. To provide a taxonomy which can help medical educators decide on the most appropriate tool(s) available to evaluate their EBM teaching, we reviewed only those tools identified as high quality against the CREATE framework [11]. The framework helps in characterising the assessments with regards to the 5-step EBP model, types and level of educational assessment specific to EBP, audience characteristics and assessment aims. The framework is meant to help developers of new tools to identify and where possible address the current gaps. Educators can assess different elements of EBM learning, and the authors of CREATE have used the work by Freeth et al. for categorising assessment of EBM educational outcomes [12].

## Results

**Fig. 1:**
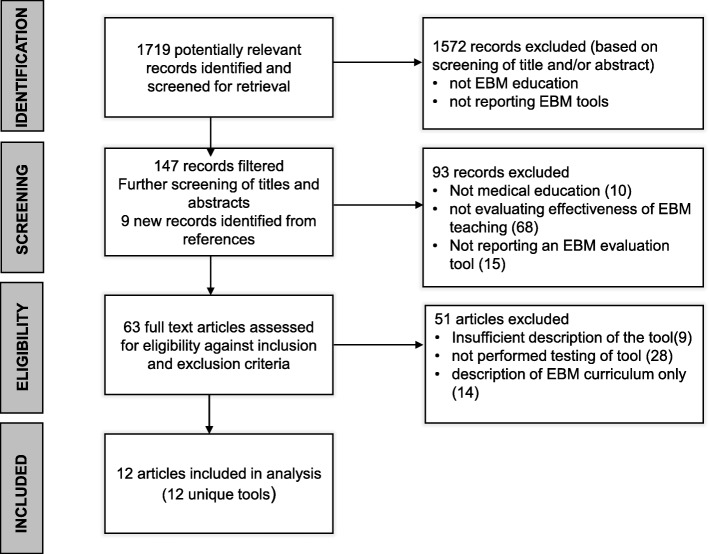
PRISMA flowchart of the systematic review

Of the 1791 articles retrieved, 1572 were excluded and 147 articles were screened for eligibility. Of these 147; 93 were excluded and 63 full text articles were identified for further screening (Fig. 1 shows the PRISMA flowchart). After assessing the 63 full text articles for eligibility against inclusion and exclusion criteria, twelve were included in the final analysis.

### Uploaded separately

The completed PRISMA checklist [13] has been attached as Additional file 2.

### Classification of tools according to the assessment of EBM practice

We categorised the twelve tools according to their relevance to the five steps of EBM. EBM step 3—‘appraise’ was the most frequently assessed using a validated tool—all twelve tools (100%) identified assessed ‘appraise’. Three evaluated the first four steps of EBM, namely ask, acquire, appraise and apply. Seven (58%) evaluated ‘ask’, seven (58%) evaluated ‘acquire’ and 4 (33%) evaluated ‘apply’. None of the seven identified evaluated the last step—‘assess’ (Table 1).

**Table 1:** Classification of tools against EBM steps evaluated

Tool	EBM steps				
	Ask	Acquire	Appraise	Apply	Assess
Taylor’s questionnaire [14]		Yes	Yes		
Berlin [7]			Yes		
Fresno [6]	Yes	Yes	Yes		
ACE [15]	Yes	Yes	Yes	Yes	
Utrecht questionnaire U-CEP [16]	Yes		Yes	Yes	
MacRae examination [17]			Yes		
EBM test [18]	Yes	Yes	Yes		
Educational prescription [19]	Yes	Yes	Yes	Yes	
Mendiola-mcq [20]			Yes		
Tudiver OSCE [21]	Yes	Yes	Yes		
Frohna’s OSCE [22]	Yes	Yes	Yes	Yes	
BACES [23]			Yes		

### Classification of tools according to the educational outcome domains measured

We have also differentiated tools according to their relevance to the seven dimensions of EBM learning, namely reaction to educational experience, attitudes, self-efficacy, knowledge, skills, behaviours and benefits to patients. Of the twelve tools, ten (83%) evaluated knowledge gain, nine (75%) EBM skills  and one (8%) evaluated attitude. None addressed reactions to EBM teaching, self-efficacy, change in behaviours or patient benefit (Table 2).

**Table 2:** Classification of tools against the seven educational outcome domains

	Outcome domains assessed by the twelve EBM instruments						
	Reaction to EBM teaching	Attitude	Self-efficacy	Knowledge	Skills	Behaviours	Patient benefit
Taylor’s questionnaire		Yes		Yes			
Berlin				Yes	Yes		
Fresno				Yes	Yes		
ACE				Yes	Yes		
Utrecht questionnaire U-CEP				Yes			
MacRae examination				Yes	Yes		
EBM test				Yes	Yes		
Educational prescription				Yes	Yes		
Mendiola				Yes			
Tudiver OSCE					Yes		
Frohna’s OSCE					Yes		
BACES				Yes	Yes		

### Quality of EBM tools and taxonomy

Quality assessment ratings are presented in Table 3. Of the twelve tools included, six (50%) were judged to be of high quality supported by established (interrater reliability (if applicable), use of objective (non-self-reported) outcome measure(s) and demonstrated multiple (≥ 3) types of established validity evidence (including evidence of discriminative validity)).

**Table 3:** High quality tools with ≥ 3 types of established validity

Tool	Reported psychometric properties							
	Content validity	Interrater reliability	Internal validity	Responsive validity	Discriminative validity	Construct Validity	Internal reliability (ITC)	External validity
Taylor’s questionnaire [14]	Yes		Yes	Yes	Yes			
Berlin [7]	Yes		Yes	Yes	Yes			
Fresno [6]	Yes	Yes	Yes		Yes			
ACE [15]	Yes	Yes	Yes	Yes	Yes			
Utrecht questionnaire [16]	Yes		Yes	Yes	Yes	Yes	Yes	Yes
MacRae [17]	Yes	Yes	Yes		Yes	Yes		

The validity assessments of the six high-quality tools used in evaluating EBM teaching in medical education are presented in Table 3. Evaluations of psychometric test properties of these tools are presented in Table 4, and their classification against the CREATE framework is presented in Table 5. The Taylor’s questionnaire [14] has a set of multiple-choice questions which assesses knowledge and attitudes and was initially validated in four groups of healthcare professionals with varying degrees of expertise (UK). It has since been assessed in a medical student cohort (Mexico). The Berlin questionnaire [7] measures basic knowledge about interpreting evidence from healthcare research and is built around clinical scenarios and have two separate sets of questions focusing on epidemiological knowledge and skills. It was initially evaluated in EBM experts, medical students and participants in EBP course (USA). The Fresno test [6] assesses medical professionals’ knowledge and skills and consists of two clinical scenarios with 12 open-ended questions. It was initially evaluated in family practice residents and faculty members (USA).

**Table 4:** Details of studies where the high-quality tools (*n* = 6) were validated for use in evaluating EBM teaching in medical education

Source instrument name and date	Instrument development-number of participants, level of expertise	EBM learning domains	Instrument description	EBM steps	Psychometric properties with results of validity and reliability assessment
Berlin questionnaire-Fritsche [7]	266 participants—43 experts in evidence-based medicine, 20 controls (medical students) and 203 participants in evidence-based medicine course (USA)	Knowledge and skills	Berlin questionnaire was developed to measure basic knowledge about interpreting evidence from healthcare research, skills to relate a clinical problem to a clinical question, the best design to answer it and the ability to use quantitative information from published research to solve specific patient problems. The questions were built around clinical scenarios and has two separate sets of 15 multiple-choice questions mainly focusing on epidemiological knowledge and skills (scores range from 0 to 15)	Appraise	Content validity Internal validity Responsive validity Discriminative validity The two sets of questionnaires were psychometrically equivalent: interclass correlation coefficient for students and experts 0.96 (95% confidence interval 0.92 to 0.98, *p* < 0.001). Cronbach’s alpha 0.75 for set 1 and 0.82 for set 2. Ability to discriminate between groups with different levels of knowledge by comparing the three groups with varying expertise: The mean score of controls (4.2 (2.2)), course participants (6.3 (2.9)) and experts (11.9 (1.6)) were significantly different (analysis of variance, *p* < 0.001)
Fresno test-Ramos et al. [6]	Family practice residents and faculty member (*n* = 43); volunteers self-identified as experts in EBM ( *n* = 53); family practice teachers (*n* = 19) (USA)	Knowledge and skills	Fresno test was developed and validated to assess medical professionals’ knowledge and skills. It consists of two clinical scenarios with 12 open-ended questions which are scored with standardised grading rubrics. Calculation skills were assessed by fill in the blank questions.	Ask, acquire and appraise	Content validity Interrater reliability Internal validity Discriminative validity Expert opinion Interrater correlations ranged from 0.76 to 0.98 for individual items Cronbach’s alpha was 0.88. ITC ranged 0.47–0.75. Item difficulties ranged from moderate (73%) to difficult (24%). Item discrimination ranged from 0.41 to 0.86. Construct validity, on the 212 point test, the novice mean was 95.6 and the expert mean was 147.5 (*p*< 0.001)
MacRae [17]	Residents in University of Toronto General Surgery Program (*n* = 44) (Canada)	Knowledge and skills	Examination consisted of three articles each followed by a series of short-answer questions and 7-point rating scales to assess study quality.	Appraise	Content validity Interrater reliability Internal validity Discriminative validity Construct validity Cronbach’s alpha 0.77 Interrater reliability—Pearson product moment correlation coefficient between clinical epidemiologist and non-epidemiologist-0.91 between clinical epidemiologist and nurse 0.78.Construct validity was assessed by comparing scores of those who attended the journal club versus those who did not and by postgraduate year of training (*p*= 0.02)
Taylor [14] Bradley et al. [24]	4 groups of healthcare professionals (*n* = 152 ) with varying degrees of expertise of EBP (UK) Group 1—with no or little prior EBP education 2—undertaken CASP workshop within last 4 weeks; 3—undertaken CASP workshop in the last 12 months; 4—academics currently teaching EBP and attended 1997 Oxford CEBM workshop Later, Bradley et al. tried with 175 medical students in RCT of self-directed vs workshop-based EBP curricula (Norway)	Knowledge and attitudes	Questionnaire 11mcqs -true, false, do not know Correct responses given 1 Incorrect responses scored 1 Do not know 0	Acquire and appraise	Content validity Internal validity Responsive validity Discriminative validity Cronbach’s alpha (0.72 for knowledge and 0.64 for attitude questions) Spearman’s correlation (internal consistency), total knowledge and attitudes scores ranged from 0.12 to 0.66, discriminative validity (novice and expert) Responsiveness (instrument able to detect change)
ACE tool- Dragan Ilic [15]	342 medical students—98 EBM-novice, 108 EBM-intermediate and 136 EBM-advanced participants (Australia)	Knowledge and skills	Assessing Competency in EBM (ACE )tool was developed and validated to evaluate medical trainees’ competency in EBM across knowledge, skills and attitudes—15 items, dochotomous outcome measure; items 1 and 2, asking the answerable question; items 3 and 4, searching literature; items 5–11 critical appraisal; items 12–15 relate to step 4 applying evidence to the patient scenario.	Ask, acquire, appraise and apply	Content validity Interrater reliability Internal validity Responsive validity Discriminative validity Construct validity—statistically significant linear trend for sequentially improved mean score corresponding to the level of training (*p*< 0.0001) Item difficulty ranged from 36 to 84%, internal reliability ranged from 0.14 to 0.20, item discrimination ranged from 0.37 to 0.84, Cronbach’s alpha coefficient for internal consistency was 0.69
Kortekaas-Utrecht questionnaire [16] (original questionnaire in Dutch, English version now available)	Postgraduate GP trainees (*n=*219), hospital trainees (*n* = 20), GP supervisors (*n*=20) academic GPs or clinical epidemiologists (*n* = 8) (Netherlands)	Knowledge	Utrecht questionnaire on knowledge on clinical epidemiology (U-CEP): two sets of 25 questions and a combined set of 50	Ask, appraise and apply	Content validity Internal validity Responsive validity Discriminative validity Content validity—expert opinion and survey Construct validity—significant difference in mean score between experts, trainees and supervisors Internal consistency—Cronbach alpha 0.79 for set A, 0.80 for set B and 0.89 for combined Responsive validity—significantly higher mean scores after EBM training than before EBM training Internal reliability—ITC using Pearson product, median 0.22 for set A, 0.26 for set B and 0.24 for combined Item Discrimination ability—median-0.35 for set A, 0.43 for set B and 0.37 for combined

*ITC* item total correlation, *RCT* randomised controlled trial, *CASP* critical appraisal skills program, UCEP Utrecht questionnaire on knowledge on clinical epidemiology for evidence-based practice

**Table 5:** Classification of the six high quality tools according to CREATE framework

Assessment category		Type of assessment	Steps of EBM				
7	Benefits to patients	Patient-oriented outcomes					
6	Behaviours	Activity monitoring					
5	Skills	Performance assessment	Fresno ACE	Fresno ACE	Berlin’s Fresno ACE MacRae	ACE	
4	Knowledge	Cognitive testing	Fresno ACE U-CEP	Fresno ACE Taylor's	Taylor’s Berlins Fresno ACE UCEP MacRae	ACE UCEP	
3	Self-efficacy	Self-report/opinion					
2	Attitudes			Taylor's	Taylor’s		
1	Reaction to the educational experience						
			**Ask**	**Search**	**Appraise**	**Integrate**	**Evaluate**

Audience characteristic: students and trainees in medical education.

Assessment aims: formative

The ACE tool [15] evaluates medical trainees’ competency in EBM across knowledge, skills and attitudes and has 15 questions with dichotomous outcome measure. It was initially evaluated with medical students and professionals with different levels of EBM expertise (Australia). The Utrecht questionnaire has two sets of twenty-five questions testing knowledge on clinical epidemiology and was initially evaluated with postgraduate GP trainees, hospital trainees, GP supervisors, academic GPs or clinical epidemiologists (Netherlands). The MacRae examination consists of three articles each followed by a series of short-answer questions testing knowledge and skills which was evaluated in surgery residents (Canada).

Details of the remaining six tools identified in this review, which did not meet the criteria for ‘high-quality’ tools are presented in Table 6. These tools have been used to evaluate EBM in medical education and assess (a) the dimension(s) of EBM learning, namely reaction to educational experience, attitudes, self-efficacy, knowledge, skills, behaviours and benefits to patients; (b) different step(s) of EBM and (c) presented results of the psychometric performance of the tool. However, they have not demonstrated multiple (≥ 3) types of established validity evidence (including evidence of discriminative validity).

**Table 6:** Details of studies which have used and validated six other tools identified as lower quality by this review for use in evaluating EBM teaching in medical education

Source instrument name and date	Instrument development, number of participants, level of expertise	EBM learning domains	Instrument description	EBM steps	Psychometric properties with results of validity and reliability assessment
Educational Prescription-David Feldstein [19]	20 residents	Knowledge and skills	Educat academic GPs or clinical ional prescription (EP)—web-based tool that guides learners through the four As of EBM. Learners use the EP to define a clinical question, document a search strategy, appraise the evidence, report the results and apply evidence to the particular patient	Asking, acquiring, appraising, applying	Predictive validity Interrater reliability Interrater reliability on the 20 EPs showed fair agreement for question formation (k= 0.22); moderate agreement for overall competence (*k* = 0.57) and evaluation of evidence (k= 0.44). and substantial agreement for searching (*k* = 0.70) and application of evidence (*k* = 0.72)
BACES-Barlow [23]	Yes postgraduate medical trainees/residents—150 residents	Knowledge, skills	BACES-Biostatistics and Clinical Epidemiology Skills (BACES) assessment for medical residents-30 multiple-choice questions were written to focus on interpreting clinical epidemiological and statistical methods	Appraisal—interpreting clinical epidemiology and statistical methods	Content validity was assessed through a four person expert review Item Response Theory (IRT) makes it flexible to use subsets of questions for other cohorts of residents (novice, intermediate and advanced). 26 items fit into a two parameter logistic IRT model and correlated well with their comparable CTT (classical test theory) values
David Feldstein-EBM test [18]	48 internal medicine residents	Knowledge and skills	EBM test—25 mcqs-covering seven EBM focus areas: (a) asking clinical questions, (b) searching, (c) EBM resources, (d) critical appraisal of therapeutic and diagnostic evidence, (e) calculating ARR, NNT and RRR, (f) interpreting diagnostic test results and (g) interpreting confidence intervals	Asking, acquiring and appraising Asking clinical questions, searching, EBM resources, critical appraisal, calculations of ARR, NNT, RRR, interpreting diagnostic test results and interpreting confidence intervals.	Construct validity Responsive validity EBM experts scored significantly higher EBM test scores compared to PGY-1 residents (*p* < 0.001), who in turn scored higher than 1st year students (*p* < 0.004). Responsiveness of the test was also demonstrated with 16 practising clinicians—mean difference in fellows’ pre-test to post-test EBM scores was 5.8 points (95% CI 4.2, 7.4)
Frohna-OSCE [22]	Medical students (*n*-26) who tried the paper-based test during the pilot phase. A web-based station was then developed for full implementation (*n* = 140).	Skills	A web-based 20-min OSCE-specific case scenario where students asked a structural clinical question, generated effective MEDLINE search terms and elected the most appropriate of 3 abstracts	Ask, acquire, appraise and apply	Face validity Interrater reliability Literature review and expert consensus Between three scorers, there was good interrater reliability with 84, 94 and 96% agreement (*k* = 0.64, 0.82 and 0.91)
Tudiver-OSCE [21]	Residents—first year and second year	Skills	**OSCE** stations	Ask, acquire, appraise and apply	Content validity Construct validity *p*= 0.43 Criterion validity *p* < 0.001 Interrater reliability ICC 0.96 Internal reliability Cronbach’s alpha 0.58
Mendiola-mcq [20]	Fifth year medical students	Knowledge	**MCQ** (100 questions)	Appraise	Reliability of the mcq = Cronbach’s alpha 0.72 in M5 and 0.83 in M6 group Effect size in Cohen’s *d* for the knowledge score main outcome comparison of M5 EBM vs M5 non-EBM was 3.54

*mcq* multiple choice question, *OSCE* objective structured clinical examination, *ICC* intraclass correlation, *NNT* number needed to treat, *ARR* attributable risk ratio, *RRR* relative risk ratio

Assessment aims: formative

## Discussion

This systematic review has identified twelve validated tools which can help evaluate EBM teaching in medical education. This review has focused on tools which used objective outcome measures, provided enough description of the tool, the EBM educational domains assessed, EBM steps assessed, and details of the psychometric tests carried out. Of the twelve tools identified, six were high-quality tools as supported by established (interrater reliability (if applicable), use of objective (non-self-reported) outcome measure(s) and demonstrated multiple (≥ 3) types of established validity evidence (including evidence of discriminative validity).

Of the five steps of EBM, ‘appraise’ was the most commonly evaluated step, followed by ‘ask’, ‘acquire’ and ‘apply’ steps. None of the tools identified evaluated the last step—‘assess’. Conducting an audit of clinical processes and outcomes and using activity diaries to document activities directly related to EBP have been suggested as possible methods of assessing EBP process [25]. Most tools evaluated knowledge and skills domains of the seven outcome domains. Few evaluated changes in attitude and behaviours. No tools were identified which could evaluate reaction to EBM teaching or the impact on patient benefit. Challenges in measuring the impact of patient benefit might be because the impact is often latent and distant and the difficulty in isolating the effect of EBM from the role of the overarching team and healthcare system on patient outcomes [8].

This is the first systematic review which has provided EBM educators in medical education a compendium of currently available high-quality tools to evaluate teaching of EBM. We have also categorised the six high quality tools identified by this review according to the CREATE framework [11] to provide a taxonomy which can help medical educators decide on the most appropriate tool(s) available to evaluate their EBM teaching. The taxonomy has categorised tools against the EBM steps and the EBM educational domains, to help developers of new tools to identify and where possible address the current gaps.

Shaneyfelt et al. [5] identified 104 unique assessment strategies in 2006, which could be used to evaluate EBP (evidence-based practice) and found that most evaluated EBM skills. In line with the present review, they also noted that of the EBP skills, acquiring evidence and appraising evidence were most commonly evaluated. Of the 104 tools identified, they categorised seven as level 1, they were supported by established interrater reliability (if applicable), objective (non-self-reported) outcome measures, and multiple (≥ 3) types of established validity evidence (including evidence of discriminative validity) [5]. The authors specifically identified the Fresno [6] and Berlin [7] as the only high quality instruments for evaluating knowledge and skills of individual trainees across the EBP steps. The 2006 review [5], however, did not categorise the level 1 tools according to the EBM educational domains assessed.

Since the 2006 review, two new tools have been identified for use in medical education with similar quality as the initial level 1 tools—ACE and Utrecht questionnaire [15, 16]. There have been more recent reviews which have included these tools—a recent review in 2013 carried out by Oude Rengerink et al [9] identified 160 different tools that assessed EBP behaviour amongst all healthcare professionals. However, the authors found that most of them subjectively evaluated a single step of EBP behaviours without established psychometric properties. They did not find any tool with established validity and reliability which evaluated all five EBP steps.

Leung et al. [26] in their 2014 review of tools for measuring nurses’ knowledge, skills and attitudes for evidence-based practice identified 24 tools, of which only one had adequate validity—the evidence-based practice questionnaire [27]. However, the authors note that the evidence-based practice questionnaire relies entirely on self-report rather than direct measurement of competence. Thomas et al. in their 2015 systematic review of evidence-based medicine tests for family physician residents found that only the Fresno test had been evaluated with more than one group of family medicine residents and had the best documentation of validity and reliability [10].

The specific focus of this review on tools used in medical education (excluding other healthcare professionals) offers unique insight and information of use to medical educators. In addition to presenting details of the identified tools, we have provided a taxonomy of tools which have been categorised according to the EBM steps evaluated and the educational outcome domains measured. We have used the qualities of level 1 category tools suggested by Shaneyfelt et al. to provide a current list of six high-quality tools and have classified them according to CREATE framework. We found that while earlier tools evaluated fewer steps of EBM and educational outcome domains, there is an increasing focus on developing more comprehensive tools which can evaluate all steps of EBM and all educational outcome domains. While most of the tools identified in this review had some validation, recent tools have had more psychometric tests performed and reported. The most recent of the tools, the Utrecht questionnaire has specifically undergone rigorous validation. The authors have carried out tests of internal consistency, internal reliability (item-total correlation), item discrimination index, item difficulty, content validity, construct validity, responsiveness, test-retest reliability, feasibility and external validation.

Similar to previous reviews [8, 10, 26], while categorising the high-quality tools against the five EBM steps, we found that the majority of validated tools focus on ‘appraise’, and fewer tools have focused on the other steps ‘ask’, ‘acquire’ and ‘apply’. There is also a need for tools which can address the last step of EBM—‘assess’. Translating research findings into clinical decisions is an important lifelong skill for healthcare professionals. EBM is not just about the ability to ask the right question, followed by searching and appraising the quality of evidence. It is bringing together clinical expertise, patient values and current best evidence into clinical decision making [1]. Multifaceted clinically integrated teaching methods along with evaluation of EBM knowledge, skills, attitudes and behaviour using validated tools can help in enhancing EBM competencies [4].

This review has identified some gaps in tools available for EBM teaching. There is a need for tools which can address all aspects of EBM steps- in particular, ‘apply’ and ‘assess’. Evidence suggests that medical education often focuses on teaching and assessing students on the first three steps of EBM—ask, acquire and appraise [8, 28]. Medical trainees should be taught how to bring together the evidence, patients’ preferences and clinical expertise in clinical decisions. As assessment drives learning, trainees should then be assessed on this step of EBM to encourage them to be lifelong learners. Secondly, within educational domains, most tools evaluate knowledge and skills with very few evaluating attitudes and behaviour. Researchers in medical education need to explore new tools which can evaluate all steps of EBM and educational outcome domains. Researchers also need to publish information on the feasibility of implementing the tools—time taken to complete and grade along with any other resource implications. This can help medical educators in making decisions about the feasibility of using these tools in assessing the effectiveness of EBM teaching. In our review, we found that while five tools had details on the feasibility of administering them, seven did not have any specific details.

This systematic review may have some limitations. We may have missed some tools, especially the ones which might have been published in grey literature. However, we searched multiple databases using a robust search strategy and screened citations from retrieved articles. Another limitation is that there may be some inaccuracies in reporting the tools against the educational outcome domains, EBM steps and validity tests. We tried to address this by having two independent reviewers extract data against the agreed checklist from the final list of articles; which was then verified by a third reviewer. Lastly our review was limited to tools used in medical education. Though literature suggests that several of these tools have also been used in other healthcare professions like nursing, dentistry and allied health professionals.

In summary, this review has helped to develop a taxonomy of the available tools based on their psychometric properties such as reliability and validity; relevance to the five EBM domains and the seven dimensions of EBM learning suggested by the CREATE framework. This will assist EBM educators in medical education in selecting the most appropriate and psychometrically validated measures to evaluate EBM teaching.

## Supplementary information

**Additional file 1.** Search strategy.

**Additional file 2.** Prisma checklist

## Data Availability

The data are available to all interested researchers upon request. Please contact the corresponding author.
