# Supplementary material for: A systematic review and taxonomy of tools for evaluating evidence-based medicine teaching in medical education
Source: Syst Rev. 2020 Apr 24;9:91. doi: 10.1186/s13643-020-01311-y (PMC7183115; doi:10.1186/s13643-020-01311-y)
Supplement: Supplementary file 1 — Additional file 1. Search strategy. [file 13643_2020_1311_MOESM1_ESM.docx]

**Additional file 1: Sample search strategy from MEDLINE**

**MEDLINE**

1. (MESH.EXACT.EXPLODE (“Evidence-Based-Medicine”))
2. (evidence-based medicine) OR EBM OR evidence based medicine
3. (“Evidence-based practice”) OR EBP OR (2evidence based practice”)
4. (MESH.EXACT.EXPLODE (“Evidence-Based Practice”))
5. (“evidence-based healthcare”) OR EBH OR (“evidence based healthcare)
6. (MESH.EXACT.EXPLODE (“Educational Measurement”))
7. (“assessment tool”)
8. (MESH.EXACT.EXPLODE (“Schools, Medical”))
9. (MESH.EXACT.EXPLODE (“Education, Medical”))
10. (MESH.EXACT.EXPLODE (“Students, Medical”))
11. (MESH.EXACT.EXPLODE (“Clinical Competence”))
12. 1 OR 2 OR 3 OR 4 OR 5
13. 6 OR 7
14. 12 AND 13 AND (pd (2005-2018))
15. 8 OR 9 OR 10
16. 14 AND 15
17. 11 AND 16
